# Applications of long-read sequencing to Mendelian genetics

**DOI:** 10.1186/s13073-023-01194-3

**Published:** 2023-06-14

**Authors:** Francesco Kumara Mastrorosa, Danny E. Miller, Evan E. Eichler

**Affiliations:** 1 Department of Genome Sciences, University of Washington School of Medicine, Seattle, WA 98195 USA; 2 Division of Genetic Medicine, Department of Pediatrics, University of Washington and Seattle Children’s Hospital, Seattle, WA 98195 USA; 3 Department of Laboratory Medicine and Pathology, University of Washington, Seattle, WA 98195 USA; 4 Brotman Baty Institute for Precision Medicine, University of Washington, Seattle, WA 98195 USA; 5 Howard Hughes Medical Institute, University of Washington, Seattle, WA 98195 USA

**Keywords:** Long-read sequencing, Genetic variation, Medical genetics, Structural variation, Mendelian disorders

## Abstract

Advances in clinical genetic testing, including the introduction of exome sequencing, have uncovered the molecular etiology for many rare and previously unsolved genetic disorders, yet more than half of individuals with a suspected genetic disorder remain unsolved after complete clinical evaluation. A precise genetic diagnosis may guide clinical treatment plans, allow families to make informed care decisions, and permit individuals to participate in N-of-1 trials; thus, there is high interest in developing new tools and techniques to increase the solve rate. Long-read sequencing (LRS) is a promising technology for both increasing the solve rate and decreasing the amount of time required to make a precise genetic diagnosis. Here, we summarize current LRS technologies, give examples of how they have been used to evaluate complex genetic variation and identify missing variants, and discuss future clinical applications of LRS. As costs continue to decrease, LRS will find additional utility in the clinical space fundamentally changing how pathological variants are discovered and eventually acting as a single-data source that can be interrogated multiple times for clinical service.

**Supplementary Information:**

The online version contains supplementary material available at 10.1186/s13073-023-01194-3.

## Background

Despite the widespread use of exome sequencing (ES) in clinical practice, approximately half of individuals with a suspected Mendelian condition remain without a precise molecular diagnosis after a complete clinical evaluation. The application of short-read whole-genome sequencing (SR WGS), while offering much more uniform coverage across the genome, has only modestly increased the solve rate [1, 2]. There are likely multiple reasons for this, including incomplete gene–phenotype associations, incomplete ascertainment of individuals undergoing genetic testing, inadequate understanding of the regulatory landscape of genes, and technical limitations of sequencing. For example, short-read sequencing (SRS), despite its accuracy, does not reliably map sequence reads to repetitive regions of our genome, such as segmental duplications, tandem repeats, or low-complexity regions enriched for GC- or AT-rich DNA [3]. There are more than one thousand protein-coding genes associated with such regions, many of which are clinically relevant, where variation is simply not reliably assayed [4]. Moreover, numerous studies over the last few years have shown that most larger, more complex forms of human genetic variation—termed structural variations (SVs) for events >50 bp in size—are missed by SRS and ES because of their association with repetitive DNA. Technological advances and new methods, thus, are critical to more fully evaluate individuals who remain unsolved after comprehensive clinical evaluation.

Although not yet clinically available, long-read sequencing (LRS) represents a promising technology to evaluate individuals with unknown genetic etiology or those who have complex changes not fully resolved by prior evaluation. Most LRS commercial platforms now routinely deliver reads >10 kbp and up to several megabases [5]. Unlike SRS, which involves amplification of DNA, LRS typically analyzes native DNA; therefore, it may be regarded as 5-base sequencing, with the ability to determine the methylation status of CpG sites in addition to the standard four nucleotides identified by SRS. Currently, LRS platforms capable of 5-base sequencing are primarily produced by two companies: Pacific Biosciences (PacBio) and Oxford Nanopore Technologies (ONT). Because the two technologies differ fundamentally in the way they generate data, leading to differences in output and error rates, it is important to consider the nuances of both when selecting which platform to use (discussed below). At the time of this writing, a synthetic long-read product is being developed by Illumina, though is not yet widely available, so it will not be discussed in this review.

Because LRS technology is relatively new, there are few carefully controlled studies comparing LRS to SRS or ES [6]. Recent work has shown that LRS technologies typically identify ~25,000 SVs per human genome in contrast to SRS of the same samples, which depending on the SV discovery tools applied, only generates 3000–10,000 [6–10]. SV discovery using SRS lacks both sensitivity and specificity making it unreliable as a clinical test. Consequently, multiple groups have shown that LRS can be used to identify disease-causing variants missed by prior clinical testing in a modest number of cases [11–17]. This increased solve rate is derived given that LRS is able to access challenging regions of the genome refractory to analysis with SRS, which simplifies variant calling and resolution of complex SVs [4, 6, 18, 19]. More than 250 medically relevant genes are more accurately ascertained using LRS-based approaches when compared to SRS [20, 21]. In particular, LRS-based approaches can resolve complex SVs [14, 22–24], repeat expansions [25, 26], and differences in methylation [15] in medically relevant regions or cases that were not solved after standard clinical testing. Finally, LRS, specifically on the ONT platform, is unique in that the data is available for analysis in near real time, which has allowed for studies showing that a complete genome could be sequenced and analyzed in less than 8 h and WGS with targeted analysis for previously known variants could be completed within 3 h [27, 28]. Together, these studies suggest that systematic application of LRS to previously unsolved Mendelian cases might increase the overall rate of diagnosis.

Here, we provide an overview of LRS technology improvements, including the advantages and disadvantages of each technology, along with the advances that have increased coverage, throughput, and accuracy. Due to the rapid developments in LRS technology over the last few years, any review of this type is likely to be soon outdated. However, we ground this assessment on existing published data and flag potential projections. Using examples from the literature, we focus on cases of Mendelian variants that were identified with LRS and refractory to analysis with ES or SR WGS. We conclude with a discussion of how LRS may be used in the clinical setting in both the near and long term, including the use of LRS as a single data source to replace most clinical testing available today.

### Long-read sequencing technologies

There are two commercially available technologies today, PacBio and ONT (Table 1), that routinely generate RNA or DNA reads greater than 10 kbp.

**Table 1:** Comparison of PacBio and ONT sequencing technologies

**Sequencing technology**	**Platform**	**Supported flow cell**	**Data production**	**Read length (kbp)**	**Mean read accuracy (%)**	**Throughput per flow cell (Gbp)**	**Estimated Cost per Gbp (US$)**^**a**^	**Applications**
Pacific Biosciences (PacBio)	Sequel II/IIe^c^	SMRT Cell 8M	HiFi	15-25 (up to 40)	>99	30-42	31-43	WGS, Gene panels, cDNA sequencing, Methylation analysis, Metagenomics and microbiome analyses
	Revio^c^	SMRT Cell 25M			>99	Up to 90-126^b^	8-11^b^	
Oxford Nanopore Technologies (ONT)	Flongle	R9.4.1	Simplex, duplex	1-100 (up to > 2000)	>95	1-2	118^d^	Amplicon or plasmid sequencing
	MinION	R9.4.1, R10.4.1			>95	15-25	29–51^e^	Amplicon sequencing, Sequencing of small genomes, Adaptive sampling, gene panel, cDNA and direct RNA sequencing, Metagenomic and microbiome analysis
	GridION	R9.4.1, R10.4.1			>95	15-25^f^	29–51^e^	
	PromethION	R9.4.1, R10.4.1			>95	100-200	6–12^g^	WGS, cDNA and direct RNA sequencing, Adaptive sampling

^a^Pricing includes exclusively SMRTbell prep and sequencing reagents run on proprietary instruments.

^b^Projected estimate: Revio was launched in Q1 2023.

^c^The Sequel IIe and Revio can process the raw sequencing data and generate HiFi reads on the instrument.

^d^Assumes output of 1.5 Gbp per flow cell using single library.

^e^Assumes output of 20 Gbp per flow cell using single library.

^f^GridION allows simultaneous sequencing on 5 flow cells.

^g^Assumes output of 150 Gbp per flow cell with three libraries.

**Fig. 1:**
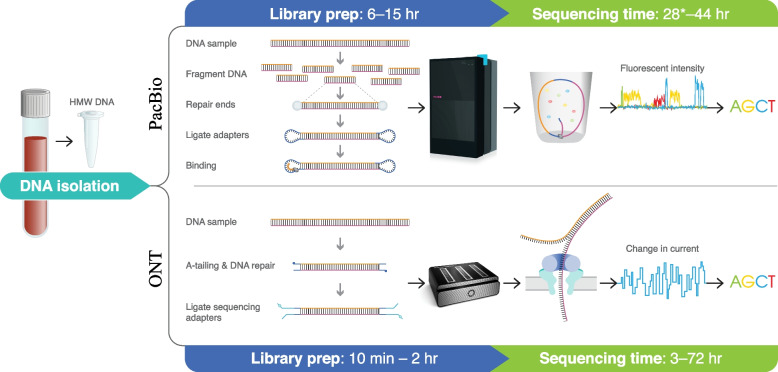
Library preparation and sequencing workflow for both PacBio and ONT. PacBio workflow: DNA is first extracted from blood or cell lines and then sheared to the desired fragment size (typically at 15–25 kbp). After DNA end repair, fragments are ligated to adapters to form circular molecules called SMRTbells. Each SMRTbell is bound by a polymerase and loaded into a single-molecule, real-time (SMRT) cell. Once the sequencing library is loaded into the SMRT cells, each SMRTbell is immobilized at the bottom of one zero-mode waveguide (ZMW). Next, fluorescently labelled deoxynucleoside triphosphates (dNTPs) are added into the wells and sequencing begins. The polymerase starts incorporating dNTP to the new DNA strand. Each incorporated fluorescent dNTP remains briefly at the bottom of the well, where a light pulse from the bottom excites the fluorophore, which is captured by a camera; the fluorophore is then released after nucleotide incorporation. Erroneous stimulation of unincorporated dNTPs can rarely occur if they are particularly close to the bottom of the ZMWs. These occurrences contribute to the error rate of PacBio sequencing. Since modified bases slightly delay the action of the polymerase, CpG methylation can be identified. *Estimate for sequencing on Revio, which has not been extensively tested. ONT workflow: DNA extraction for ONT sequencing can depend on the desired read lengths and may be either a column-based or other extraction. Quality control steps could include an assay to evaluate contamination from the DNA extraction step and recovered fragment length. For DNA sequencing, libraries are typically prepared using either a rapid transposase-based kit, or a longer ligation-based prep that preserves fragment lengths. Libraries are loaded on the flow cell and run for the desired amount of time, with washes as needed based on flow cell performance. Sequencing data can be base called on the machine or transferred to a remote host for processing

The technologies differ radically in how sequence data are generated (Fig. 1). PacBio sequencing depends upon a DNA polymerase tethered to the bottom of a well of picolitre volume known as a zero-mode waveguide (ZMW) (Fig. 1). Here, the DNA polymerase associates with a single molecule of native DNA incorporating fluorescently labelled deoxynucleoside triphosphates (dNTPs) as it polymerizes. The action of the polymerase liberates the fluorescently labelled phosphates allowing successive nucleotide incorporations to be directly assayed by a set of precisely positioned lasers and CCD cameras. The sequence data, as a result, has been referred to as single-molecule, real-time (SMRT) sequencing. PacBio offers two sequencing modes. The original, called continuous long-read (CLR) sequencing, was designed for maximizing the length of the sequence reads and typically involved the preparation of libraries greater than 30 kbp in length. In this case, the DNA polymerase typically passes through the DNA molecule only once, generating one single-pass read with a typically high error rate resulting in a read accuracy of ~85–92% [5].

The second sequencing mode, introduced in 2019 [29], uses high-fidelity (HiFi) reads (also referred to as circular consensus sequencing (CCS)) and, as the name suggests, is designed for accuracy instead of length. It works by targeting shorter fragments of DNA (10–30 kbp) and ligating a hairpin adapter (termed a SMRTbell) at both ends of the DNA fragment creating a circular molecule. As a result, the polymerase iterates through the reverse and forward strand of the molecule multiple times generating individual subreads (Fig. 1). These reads are combined, to generate a highly accurate consensus sequence that is estimated to be >99.9% accurate (QV >30). As a result of this CCS, HiFi sequencing is currently the most accurate LRS technology but is limited to comparatively shorter library sizes. The shorter the insert, the more accurate the consensus sequence that is generated because of an increase in the number of iterations. Because modified bases pass more slowly through the polymerases than non-modified bases, CpG methylation can be deduced from dwell time in the polymerase [30]. Given the advantage of highly accurate reads, PacBio is currently focused on HiFi production and CLR sequencing is considered outdated.

Base calling is the first step needed to convert the raw sequencing data into a nucleotide sequence. In SMRT sequencing, as each nucleotide is incorporated by the polymerase, the fluorescent signal is recorded. The first base-calling step converts each fluorescent pulse into a base, generating a single long read (Fig. 1). This long read is then separated into subreads, each corresponding to a single polymerase pass through the DNA molecule. The alignment of subreads eventually generates a highly accurate consensus sequence. This correction method is allowed by the stochastic nature of PacBio errors, which decreases the possibility of having the same error in multiple subreads. Thus, discrepancy between subreads can be corrected with sufficient sequence coverage. Base calling is computationally intense; hence, the latest machines are capable of outputting CCS reads directly (Table 1). With the introduction of CCS, PacBio sequencing accuracy has become comparable to that of Illumina with the majority of residual errors confined to indels in homopolymers [29].

There are three different PacBio sequencing machines currently in use. The Sequel system (released in 2015) provides the lowest throughput, supporting SMRT cells with 1 million ZMW. It was originally designed for CLR sequencing and then adapted for HiFi. The Sequel II (released in 2019) and the Sequel IIe (2020) systems provide much higher throughput. Both support 8 million ZMWs (8M SMRT cells) and are optimized for HiFi sequencing. The Sequel IIe provides increased computational capacity compared to the previous model, which facilitates more rapid HiFi production and data processing. The Sequel II systems have become the current standard for SMRT sequencing in research laboratories. In Q1 2023, PacBio released a new machine called Revio, with capacity for 100 million ZMW (4 × 25 million reactions). The new design promises a 15-fold increase in throughput and a 4-fold reduction in cost with the potential of sequencing ~1300 human genomes per year. Since the Revio has not gone through extensive test and validation yet, we limit subsequent discussion to the Sequel II and IIe systems.

ONT sequencing, unlike most other sequencing technologies, does not depend on the action of DNA polymerase but rather an unwinding enzyme and pore protein that effectively threads single-stranded DNA or RNA molecules through a pore across a charged synthetic membrane (Fig. 1). As the molecule passes through the pore, changes in conductance are detected and are characteristic of particular nucleotide compositions. As a result, the sequence of the DNA or RNA molecule can be inferred. Library preparation is achieved through one of two methods. A rapid protocol exists and can be completed in approximately 10 min, with the drawback that random integration of adapter libraries shortens DNA fragments prior to sequencing. A second ligation-based protocol preserves the DNA fragment length and can be completed in approximately 1–2 h. In both cases, libraries are loaded onto a flow cell and can be run for as long as 72 h. Pores become unavailable over time; thus, the output of a sequencing run can be improved by washing and reloading of new libraries during the sequencing experiment. Methylation can also be determined based on differences in the current profile.

Similar to PacBio sequencing, raw sequencing output from the ONT machines has to be converted into nucleotide sequence through a base-calling process (Fig. 1). The current software used for ONT base calling is Guppy, which employs a recurrent neural network to determine sequence from raw signal. The speed and accuracy of base calling depends on which model is used, either “fast,” “high accuracy,” or “superior.” Because base calling is a computationally intensive process (most often performed on powerful graphical processing units (GPUs)), some users prefer a less accurate model that will complete quickly, such as the fast model (85–92% median read identity [31]). Alternatively, users who value accuracy over speed may choose the superior model (92–96% median read identity [31]). While several factors determine how much slower the superior model is than the fast model for a particular sample, our experience is that the superior model can be at least 10 times slower than the fast model on a high-end NVIDIA GPU. Methylation can be called concurrently by Guppy if a model trained to detect 5mC is used, resulting in slightly longer base-calling times and a slight improvement in base-calling accuracy. Changes to the signal file format and improvements to the base-caller architecture are anticipated that are likely to significantly decrease the amount of time and computational resources required for base calling.

One criticism of ONT sequencing in the past has been its lower accuracy when compared to SRS or PacBio HiFi. Improvements in chemistry, pore design, and base-calling models have increased per-read accuracy over time, with current single-nucleotide variant (SNV) recall at 30× coverage of 99.4%, and indel recall of 63–68% [32]. Indel recall increases only modestly as coverage increases, rising to 73–78%, for example, at 60× coverage [32]. There is not a well-described sequence bias in ONT sequencing as has been observed for HiFi PacBio data, which biases against regions enriched in GA/TC repeats [33]. However, a recent analysis showed that ONT is prone to base-calling errors for telomeric repeats and repeats that are represented by similar current profiles [34], while these errors are not present in equivalent PacBio sequences. Also, ONT does struggle to accurately resolve homopolymers longer than 5–7 nucleotides as the dwell time for a set of identical nucleotides in the pore is difficult to accurately determine [5]. Recently, ONT introduced a new pore, known as R10, which has a longer pore head, resulting in higher accuracy reads, with improvements in calling indels in homopolymers [35, 36].

There are several unique aspects of ONT sequencing. First, individual pores can be computationally controlled via software in real time—a sequencing mode known as adaptive sampling. This method works because signal from individual pores is sent to the controlling computer in real time allowing immediate base calling and alignment to a reference genome [37]. Therefore, during sequencing, it is possible to determine if the particular sequence maps to a region of interest. If not, the current at the pore can be reversed, the DNA molecule ejected, and a new molecule will begin sequencing. In this way, specific regions of the genome can be enriched or depleted during sequencing. Enrichment using adaptive sampling depends on several variables, such as fragment length, size of reference genome, and the ONT machine used. As an example, sequencing of a human genome with 10 kbp average fragment sizes results in 4–6× enrichment on a GridION over the region of interest [15]. Adaptive sampling recently became available on the PromethION [38] but has not been widely tested to determine its performance compared to the GridION. While the ONT platform, like PacBio, supports sequencing of complementary DNA (cDNA), another unique aspect is the ability to directly sequence native mRNA molecules using dedicated kits. This allows direct measurement of the length of a poly-A tail and, in principle, direct detection of mRNA modifications. Detecting RNA modifications using ONT sequencing is an emerging field of research, as more than 150 modifications are now known, but only a few can be reliability detected with current methods [39, 40]. Sequencing of other types of RNA molecules, such as tRNA, is an active area of research [41].

Multiple ONT sequencing platforms exist, with the PromethION being the largest device offered in either a 24- or 48-flow cell configuration (Table 1). Because a PromethION flow cell is capable of sequencing a human genome to 30–40-fold sequence coverage over a 72-h run with multiple washes, a single PromethION with 48 channels could sequence up to 98 human genomes per week. The GridION is a smaller physical device that is capable of sequencing five MinION flow cells simultaneously. Adaptive sampling is commonly performed on the MinION, and an adapter allows Flongle flow cells to be run here as well. The MinION, the smallest physical sequencer, is smaller than a typical stapler and can run both MinION and Flongle flow cells. A unique feature of ONT sequencing is portability in that the smaller devices, such as the Flongle or MinION, can be powered by a laptop, allowing them to be used in isolated areas or in resource-limited settings [42–44], and even in extremely remote locations, such as Antarctica [45] and the International Space Station [46].

Several polishing tools have been developed to improve the error rate of both PacBio CLR and ONT. They can be divided into hybrid tools, which combine SRS and LRS data, such as Hercules [47], proovread [48], LoRDEC [49], CoLoRMap [50], HG-CoLoR [51], and HALC [52]; and self-correction-based tools, such as FLAS [53] and LoRMA [54]. A recent comparison of several correction tools revealed that hybrid tools outperform nonhybrid methods, particularly when LRS coverage is low [55]. One downside of these polishing algorithms is the time they require for correcting LRS data, potentially requiring multiple days for a genome smaller than humans [55]. Therefore, more efficient methods will be required in the future to see these tools more routinely used. Alternatively, novel approaches, such as the newly released variation graphs-based tool called VeChat [56]; or DeepConsensus [57] from PacBio and Google, which use a deep-learning approach. DeepConsensus is available on the new Revio and is likely to improve error correction without the need for orthogonal data or additional compute resources.

## Targeted long-read sequencing approaches

Because of the higher costs of LRS, there has been interest, especially early on, in developing and evaluating targeted long-read sequencing (T-LRS) methods. The simplest strategy is to use PCR to amplify a region or multiple regions of interest. This results in an over-enrichment of high-priority regions, with the disadvantage of loss of methylation information during the amplification process. In addition, it can be challenging to reliably amplify regions larger than 10 or 20 kbp requiring tedious optimization and primer redesign. As a proof of the efficacy of this approach, Loomis et al*.* showed that SMRT sequencing of long PCR products of *FMR1* from patients with Fragile X syndrome allowed characterization of trinucleotide repeat expansions in patients with up to 750 repeats, an unachievable goal for short-read-based approaches [58].

Another T-LRS approach is hybridization capture. Typically, DNA is first sheared, and the DNA fragments are preselected according to the desired insert size (either <1 kbp or >1 kbp) [59]. The fragments containing regions of interest are then selected using a hybridization-based target enrichment kit. Once again, this step requires PCR amplification of the selected fragments to achieve sufficient DNA quantity for library preparation resulting in a loss of methylation signal and the amplification biases associated with PCR. Nevertheless, Wang and colleagues demonstrated the usefulness of this method by sequencing and characterizing a locus associated with reciprocal recurrent rearrangements associated with Potocki-Lupski syndrome (PTLS) and Smith-Magenis syndrome [59]. In three patients with PTLS, both known and novel breakpoints were characterized, which mapped within segmental duplications driving these rearrangements. Hybridization capture methods allow isolation of specific fragments of DNA, which could be theoretically sequenced on both PacBio and ONT instruments. However, ONT efforts are more focused on a computational method to sequence only specific regions of the human genome without prior sample treatment. This method will be discussed later.

To overcome limitations associated with PCR-based approaches, alternative strategies have been developed. CRISPR/Cas9-based target enrichment, for example, starts with a dephosphorylation step then uses an RNA-guided Cas9 digestion to expose new phosphorylation sites. The sequencing library then only is ligated to those molecules with free 5′ phosphorylation sites [60]. The CRISPR/Cas9-mediated approach was first validated by evaluating trinucleotide repeat expansions in individuals with Huntington’s disease (CAG repeats in *HTT*) and Fragile X [61]. Variations on this basic approach have been recently developed, including methods that perform digestion of dsDNA molecules not protected by Cas9 enzyme, and separate DNA molecules after cutting using pulsed-field gel electrophoresis (PFGE) [62, 63]. This approach has been successfully implemented on both the PacBio and ONT platforms. For example, Gabrieli and colleagues used Cas9-Assisted Targeting of Chromosome segments (CATCH) to target and sequence *BRCA1* and its flanking regions on an ONT platform [62]. Instead, Walsh and colleagues designed guide RNA that targeted the *BRCA1* and *BRCA2* loci and utilized PacBio to sequence the fragments [63]. Both studies isolated the DNA fragments of interest with gel electrophoresis, but Gabrieli et al*.* used DNA amplification prior to sequencing (possibly due to a low number of isolated DNA).

Even though CRISPR/Cas9-mediated protocols have been successfully used in recent studies, difficulty in designing guide RNA that result in high yield have limited widespread adoption. Indeed, PacBio withdrew official support for such CRISPR/Cas9-mediated workflows in 2021. Currently, PacBio collaborates with Twist Biosciences, which offers hybridization capture-based panels: one targets 389 genes (~20 Mbp) difficult or impossible to fully characterize with SRS; a second covers 49 genes (2 Mbp) important for drug metabolism and therapeutic response; and it is also possible to design a custom panel. As previously discussed, these panels will not preserve methylation status, since DNA amplification is necessary.

Adaptive sampling in conjunction with ONT can be used to enrich or deplete specific regions of a genome during sequencing. This strategy has been successfully used for both human and nonhuman applications. It is strictly computational in nature requiring no additional experimental setup. It has been used to characterize multiple loci with repeats commonly associated with human disease, phasing of pathogenic variants, and characterizing complex rearrangements [15, 64, 65]. The decision to perform T-LRS over WGS is typically driven by cost, as smaller regions of the genome can be currently evaluated more inexpensively than the entire genome. It is also particularly useful in solving recessive cases of Mendelian disease when only one of the two pathogenic variants has been discovered and multiple cases can be multiplexed [15, 37, 38]. Moreover, at the end of last year, a T-LRS-based workflow was designed to target 59 loci associated with repeat expansion diseases and facilitate downstream data analysis [66]. As the cost of LRS continues to drop, it is likely that the use of T-LRS will wane and WGS will become the dominant technology for variant discovery. In our experience, we have moved away from T-LRS in favor of WGS approaches to assess other loci more comprehensively, including modifier loci, more uniformly. For all targeted sequencing approaches, it is important to remember that they depend on *a priori* knowledge of the disease-associated loci.

## Quantity and quality of input DNA/RNA for long-read sequencing

LRS requires high molecular weight (HMW) DNA composed of long fragments and a higher input quantity compared to SRS. For optimal library preparation and sequencing, PacBio protocols ideally require 90% of fragments to be >10 kbp long and 50% to be >30 kbp long. 1 µg of HMW DNA is required for SMRT Cell 8M (Sequel II/IIe) and 2 µg for SMRT Cell 25M (Revio) (see PacBio website for complete protocols). ONT protocols require the amount of small fragments (<20 kbp) in the DNA sample to be the lowest possible, as shorter fragments would be preferentially sequenced. The minimum size threshold can be determined according to the purpose of the experiment, but to take full advantage of LRS, most DNA fragments should surpass at least 30-40 kbp (no theoretical upper limit for ONT read length). ONT protocols require 1.5-3 µg of input HMW DNA, and a low-input protocol, which requires a PCR step, is also available (see ONT website for complete protocols). This is optimal for certain conditions, but base modification signals will be lost during amplification and reads will be comparatively shorter. For both technologies, input DNA quality can be improved with a size selection aimed to remove shorter fragments, but this procedure requires a higher initial DNA amount because some will be lost during the process. HMW DNA for LRS should be extracted from fresh blood or cell pellets. Typically, 10 million cells or 500 µl of blood are sufficient to obtain 100-125 µg and 10-35 µg of DNA respectively using commercially available HMW DNA extraction kits.

ONT also has a protocol for ultra-long (UL) libraries. In this case, HMW DNA should be extracted with a dedicated phenol-chloroform-based protocol [67–69]. For UL libraries, the input DNA ranges from 20 to 40 µg. For both the technologies, older DNA extractions and samples that have been frozen and defrosted multiple times are less ideal for LRS due to DNA damage and fragmentation.

Both the ONT and PacBio Sequel II/IIe platforms are capable of transcriptome sequencing and can perform bulk and single-cell cDNA sequencing with different kits. Bulk sequencing using PacBio requires 300 ng of RNA with RNA integrity number (RIN) ≥7 while ONT requires 200 ng of total RNA for cDNA sequencing and 500 ng for direct RNA sequencing. Single-cell sequencing requires between 15 ng and 60-75 ng of cDNA, with the PCR cycles in the protocol adjusted according to the amount of starting material. cDNA sequencing is currently unavailable on Revio, but dedicated kits are expected in the near future (see Other Applications for more information).

## Analysis of long-read sequencing data

There are two basic approaches to identify variants using LRS. Like SRS, the most straightforward approach is read based—i.e., mapping the reads against a reference genome. Because read lengths are typically longer than most common repeat sequences (>10 kbp), the approach dramatically increases the sensitivity for SV detection. The first LRS-based studies reported >20,000 SVs per human sample [6, 7, 19], markedly higher than early data based on SRS (such as the 1000 Genomes Project), which reported only 2100–2500 SVs per genome [70] after rigorous filtering. Applying multiple SRS SV callers increases this number; for example, gnomAD-SV contains SV calls from SRS data of ~15,000 individuals and reported a median of 7,439 high-quality SVs per genome [8]. Read-based mapping approaches using LRS have improved with the release of specialized alignment tools optimized to handle longer and more error-prone data (BLASR [71], MHAP [72], NGMLR [73], and Minimap2 [74]) and software dedicated to variant discovery and phasing (WhatsHap [75], DeepVariant [76], Sniffles [73], PBSV [29], Phased-SV [6], and CuteSV [77]). While these tools continue to rapidly evolve, Minimap2 is particularly valuable for the alignment of large segments of DNA to define the breakpoints of large structural variants. DeepVariant shows excellent sensitivity for the discovery of SNVs while Sniffles and PBSV are considered current state of the art for the discovery of structural variants. LongPhase [78] can complement the analysis with variant phasing.

**Fig. 2:**
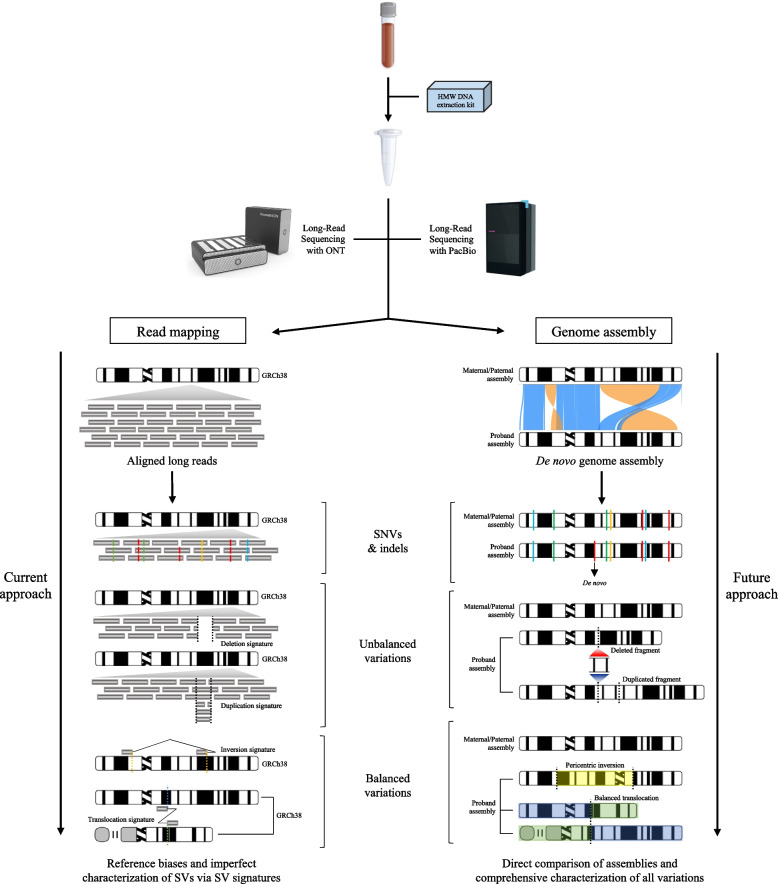
Read mapping versus de novo genome assembly for variant discovery. A traditional approach uses long-read mapping to a reference genome to identify SNVs, indels, and SV signatures, while de novo genome assembly reconstructs the two haplotypes of the sequenced individual and permits the direct comparison of assemblies (in clinical settings, ideally, parents versus proband). Genome assembly improves variant discovery, as all types of variations are fully sequence resolved and do not have to be inferred from SV signatures. Moreover, using a reference genome such as GRCh38 introduces biases due to the incompleteness of certain regions and misassembled complex loci. De novo genome assembly is the approach that we expect to substitute all the others and eventually be the gold standard method for variant discovery. (Visualization of assembly comparison adapted from SafFire [90].)

Unlike SRS, longer reads also enable reliable assembly-based discovery of variants. In principle, de novo genome assembly of long-read datasets has the potential to determine the complete or nearly complete telomere-to-telomere (T2T) DNA sequence of both haplotypes of a sample [4, 79, 80]. Recently, several genome assemblers have been developed for this purpose, such as HiCanu [33], Peregrine [81], wtdbg2 [82], Flye [83], Shasta [84], hifiasm [85, 86], and Verkko [87]—the latter is a hybrid assembly approach that combines the scaffolding potential of ONT with the high accuracy of HiFi. Genome assembly provides the most complete representation of a human genome and the potential to investigate the full spectrum of human genetic variation ranging from SNVs to fully sequence-resolved SVs, including copy number variants [88] (Fig. 2). Although close, complete T2T assembly of a diploid genome has yet to be achieved because of the challenges of traversing complex repetitive regions associated with acrocentric, centromeric, or segmentally duplicated DNA [79, 88]. The key to the assembly-based approach is correctly separating the long reads into the two constituent haplotypes underpinning each diploid genome. Over the last two years, two basic strategies have emerged depending on either the use of parental SR WGS data for trio-binning [89] or physical-based approaches where parental data are unavailable. The latter takes advantage of single-cell strand sequencing data (Strand-seq) [10] or high-throughput capture chromatin conformation (Hi-C) data [86] to identify SNV haplotypes obtained from SRS data from the same sample to then effectively phase LRS data and assembled contigs. Both methods effectively allow SNVs to be physically phased on a particular homologous chromosome. Strand-seq depends on replication and BRDU incorporation followed by degradation of the newly synthesized strand and single-cell sequencing technology to phase SNVs on the template strand for each chromosome; while Hi-C depends on crosslinking and proximity ligation to define SNVs and therefore build up locally phased haplotypes. This information is used to phase long-read sequences and assembled contigs to generate T2T chromosomes at the chromosomal level.

In 2021, the Human Genome Structural Variation Consortium (HGSVC) successfully assembled haplotype-resolved genomes of 32 human genome samples (64 haplotypes) sequenced with both CLR, HiFi PacBio, and Strand-seq as phasing technology. The authors developed a phased assembly variant (PAV) caller that enabled, for the first time, variant discovery (SNVs, indels, SVs) by direct comparison of two haplotypes of a single sample against the human reference genome. This study identified more than 100,000 SVs in the general human population providing the first comprehensive sequence-resolved map of human genome structural variation in linkage disequilibrium with flanking SNVs facilitating the discovery of new expression quantitative trait loci and disease associations [9]. Importantly, once linkage disequilibrium and breakpoints of common SVs were fully resolved, the analysis showed that new genotyping tools (e.g., PanGenie) [91] could be employed to go back to existing SRS datasets to make new associations. More than a year later [92], the Human Pangenome Reference Consortium (HPRC) assembled a more complete pangenome from 47 human genomes (94 haplotypes) using HiFi PacBio and parent–child Illumina WGS data. While not yet complete, the SV catalogs (as well as the underlying pangenomes) produced by the HGSVC and HPRC are providing a useful roadmap of “normal” human genetic variation to help focus on potentially pathogenic variants in human disease samples.

**Fig. 3:**
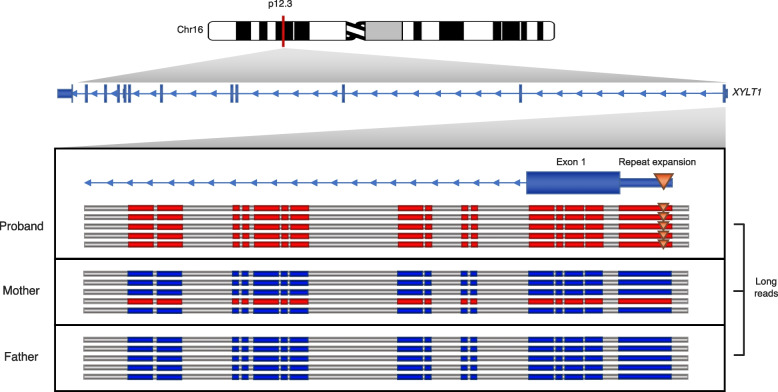
Pathogenic GGC repeat expansion in the 5′ untranslated region (UTR) of *XYLT1.* This variation was characterized in a patient known to have Baratela-Scott syndrome caused by expansion of a maternal premutation allele and paternally inherited deletion [15]. The expansion leads to hypermethylation (red) of the UTR and surrounding area. The father did not carry the expansion; however, some level of methylation was detected in the mother, who was heterozygous for a premutation allele

In addition to increased sensitivity for variant discovery, the sequencing of native DNA as opposed to amplified material (e.g., bridge amplification Illumina) has meant that methylation, and other modifications of the native DNA, may be determined (Fig. 3). Both PacBio and ONT have developed specialized tools: Primrose [93] uses a convolutional neural network to predict the 5-Methylcytosine (5mC) of CpG dinucleotides from polymerase kinetics during sequencing, while Nanopolish [94] uses a pre-trained hidden Markov model to distinguish 5mC from unmethylated cytosines based on subtle changes in the current. However, many other tools dedicated to 5mC detection and other base modifications were developed for ONT data, such as Tombo/Nanoraw [95], DeepSignal [96], DeepMod [97], and Megalodon [98], albeit now methylation detection is built into the current ONT base-caller, Guppy [99]. These methods circumvent some of the drawbacks of bisulfite sequencing (considered so far, the gold standard method for methylation analysis), such as protocol complexity, DNA degradation caused by bisulfite treatment, and read mapping limitations.

Simultaneous methylation and genetic variant characterization are particularly relevant to the study of human disease. Pathogenic repeat expansions, for example, are frequently associated with hypermethylation of the promoters and their genes leading to the loss of expression [25, 100, 101]. Moreover, individuals with pathogenic repeat expansions but showing leaking transcription/translation and possibly less extensive hypermethylation are often less severely affected [102–104]. Recently, Miller *et al.* 2021 confirmed that a known case of Baratela-Scott syndrome caused by a repeat expansion and associated hypermethylation could be evaluated by T-LRS and methylation analysis with Nanopolish. Notably, the authors showed that hypermethylation was detected for the premutation allele carried by the mother, a level of detail not achievable with prior methods (Fig. 3). With respect to cancer, methylation characterization is key. Different methylation profiles are frequently associated with different cell types and the pathogenic properties of various tumors often associate with methylation of tumor suppressor genes [105–107]. In such cases, it is critical that relevant tissues be ascertained for methylation and somatic changes.

## Other applications

Outside of strict genetic variant discovery, LRS has provided new biological insights and opportunities related to health more broadly. ONT has the potential to sequence both cDNA [108] and native RNA molecules [109]. While both provide insight into the structure of longer isoforms and full-length transcripts, the latter does not involve conversion to cDNA or subsequent amplification so there is the potential to directly assess RNA modifications. This method, called direct RNA sequencing, has been used to study RNA from bacteria and viruses [110–113] as well as that of humans [114, 115]. However, as recently discussed by Jain *et al.*, some limitations still prevent widespread use of this technology [116]. For instance, direct RNA sequencing requires a high amount of starting material, long RNA transcripts are underrepresented, and base-calling accuracy is below that of DNA sequencing. Thus, direct RNA sequencing may not be sufficient to accurately identify all open reading frames and splice sites [116].

In contrast, PacBio is limited to cDNA sequencing, and its full-length isoform sequencing protocol, termed Iso-Seq, has successfully been used to characterize splicing events, detect fusion genes, and identify tissue- and allele-specific isoforms both at the bulk [117–120] and single-cell/single-nuclei level [121–124]. One limitation of PacBio Iso-Seq is its limited output, which substantially increases the cost of this assay. For this reason, a new protocol called single-cell MAS-ISO-seq was recently developed by PacBio in collaboration with 10X Genomics and the Broad Institute [125]. This method concatenates single-cell cDNA molecules generated by 10X Genomics technology into single fragments that can be used for LRS, increasing throughput by >15-fold. Given the decrease in cost and additional information regarding isoforms that this new method allows, in February 2023, it was announced that MAS-ISO-seq will be soon adapted for bulk sequencing.

In addition to RNA sequencing, LRS has also complemented SRS studies of the microbiome and provided several critical advantages. Chen *et al.* showed that the use of the two technologies improved microbe genome assembly and SV detection (particularly for insertions and inversions) [126]. The study also demonstrated how microbial SVs could be used as a fingerprint and track the flora of the gut microbiome given the high structural diversity between individuals. Further applications, among others, include the study of virus-human integration [127] and virus surveillance [128, 129], including SARS-CoV-2.

## Human pathogenic variant discoveries

The number of reports using either PacBio or ONT to successfully identify pathogenic variants missed by clinical testing has dramatically increased over the last few years [11–17, 23–26, 130, 131]. While large-scale and systematic studies evaluating the power of LRS to identify variants in unsolved cases are lacking, efforts to date have suggested that one of the biggest gains will be the resolution of disease-associated SVs. An early study showed that 85% of common SVs had not been reported in previous large SRS studies [18]. Specifically, 92% of insertions and 69% of deletions were novel. Since deletions and insertions are variants of large effect and a known cause of genetic disease [132], this report suggested that the application of LRS would be beneficial to the study of unsolved Mendelian cases, particularly those with negative ES and SR WGS. Several groups have subsequently applied LRS to detect SVs missed or not fully clarified with ES or SR WGS (Table 2).

**Table 2:** Example disease-associated variations resolved by LRS; additional examples in Additional file 1: Table S1

**Variant class**	**Associated disease - locus of interest**	**Long-read sequencing technology**	**Previous approaches**	**Details**	**Citation**
Trinucleotide repeat expansion	Neuronal intranuclear inclusion disease, oculopharyngeal myopathy with leukoencephalopathy, oculopharyngodistal myopathy	HiFi WGS	SR WGS	Characterization of trinucleotide repeat expansion in a candidate locus	[25]
Single-nucleotide variant	Angelman syndrome	T-LRS ONT	SR WGS	Identification of the parent of origin of a pathogenic *de novo* variant	[133]
Structural variations	Duchenne muscular dystrophy	ONT WGS	T-SRS, T-LRS	Identification and characterization of a pathogenic complex SV	[13]
	Hereditary cancer	ONT WGS	SR WGS	Reinterpretation and characterization of SVs in cancer patients	[134]
	Retinitis pigmentosa	ONT WGS	T-SRS	Identification of two likely pathogenic SVs	[17]
	Complex β-globin genes with SV clusters	T-LRS HiFi	MLPA	Haplotype characterization of complex SV-rich loci; breakpoint characterization of deletions, inversions, and duplications	[22]

**Fig. 4:**
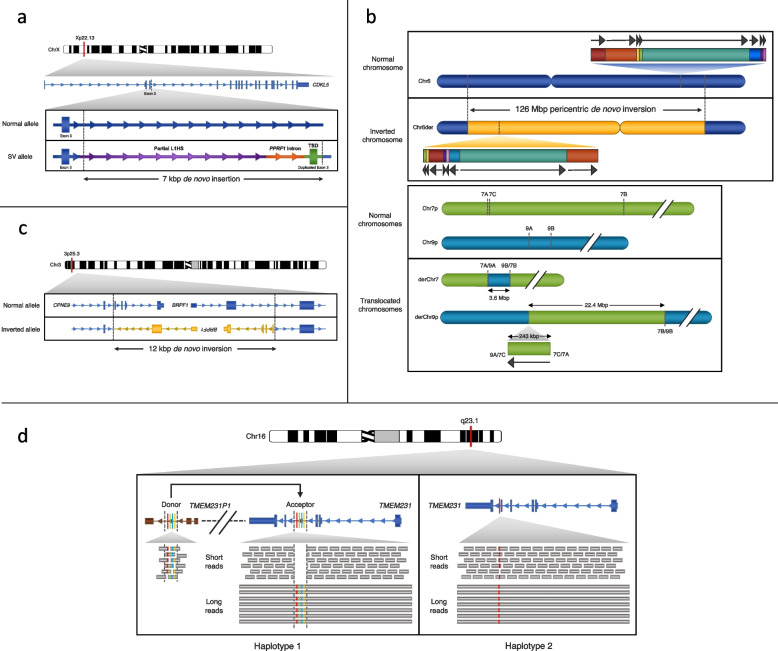
Examples of SNVs and SVs characterized by LRS that were difficult to fully resolve using SRS. **a** De novo insertion containing an additional copy of *CDKL5* exon 3 identified by Hiatt et al*.* in an individual with intellectual disability [14]. **b** Complex chromosomal rearrangement including a 126-Mbp pericentric chromosome 6 inversion that contained a 9.3-Mbp region composed of eight segments rearranged in position and orientation identified by Hiatt et al*.* in an individual with intellectual disability [14]. The same individual also carried two insertional translocations between chromosomes 7 and 9. **c** De novo inversion predicted to disrupt both *CPNE9* and *BRPF1* identified by Mizuguchi et al*.* in two monozygotic twins with Dravet syndrome [16]. **d** A gene conversion identified by Watson et al*.* in a fetus with Meckel-Gruber syndrome that did not map well by SRS [12]. LRS identified a likely pathogenic SNV in the intron 5 splice donor site (G, red; haplotype 2) of *TMEM231* in *trans* with a cluster of four missense SNVs (G, red; A, green; C, blue; and T, yellow; haplotype 1)

Hiatt and colleagues, for example, evaluated six probands with neurodevelopmental disorders and their unaffected parents using PacBio sequencing [14]. Analysis of the LRS data revealed a de novo ~7 kbp insertion (Fig. 4a) in *CDKL5* in one proband (n. 6) and a complex de novo SV in a second patient (n. 4) (Fig. 4b). Analysis of the LRS data allowed the authors to fully characterize the insertion as a ~4.3 kbp 5′ truncated, retrotransposed L1 repeat (including a poly[A] tail) with ~2.6 kbp of sequence identical to an intron of the nearby gene *PPEF1*, and a 119 bp target-site duplication that included a copy of exon 3 from *CDKL5*. The presence of the duplicated exon 3 was predicted to cause a frameshift in the transcript and inclusion was confirmed by RT-PCR. *CDKL5* was previously associated with early infantile epileptic encephalopathy 2 (OMIM #300672), a condition that overlapped with the proband’s phenotype. In proband 4, a large complex de novo SV affecting chromosomes 6, 7, and 9 was identified. Examination of the proband’s haplotype-resolved genome assembly revealed a 126-Mbp pericentric-inverted fragment with eight additional breakpoints and eight rearranged fragments inside, some of which were inverted (Fig. 4b). Six genes were predicted to be disrupted by the presence of the 10 breakpoints on chromosome 6, but the region was not previously associated with neurodevelopmental disorder phenotypes. In the same sample, two translocations between chromosomes 7 and 9 were identified with part of the translocated region of chromosome 7 also inverted. The expected effect of this complex rearrangement is the disruption of *DGKB* (chromosome 7) and *MLLT3* (chromosome 9). A decreased transcription of *MLLT3* was observed from qPCR data, while *DGKB* could not be tested due to its low level of expression in blood. Balanced translocations involving chromosomes 4 and 9 and disrupting *MLTT3* were previously reported in patients with phenotypes partially overlapping that of the proband [135, 136].

These examples highlight the complex nature of some of the genetic variants underlying disease that are difficult to detect or fully resolve using SRS. While these events represent promising candidates, they are not easily classified under existing American College of Medical Genetics (ACMG) recommendations [137]. As sequence-resolved complex rearrangements become more commonplace, additional updates to ACMG guidelines will be required that take into account events involving multiple breakpoints that likely alter transcriptional and regulatory control. It is reassuring that retrospective analysis using SRS data shows, in fact, that orthogonal sequence platforms confirm most of the new breakpoints being identified by LRS. However, discordance among SRS calling tools, incorrect or incomplete classification of size and class of variants, and the stringent requirement to minimize false positives have made discovery using SRS data extremely challenging. Such increased resolution for complex pathogenic events, including sequence resolution of cytogenetic/karyotypic rearrangements [15], is why long-read WGS (LR WGS) represents a potential alternative to SR WGS as a single clinical test.

Another recent compelling study by Mizuguchi and colleagues evaluated two monozygotic twins suspected to have Dravet syndrome with negative ES [16]. HiFi WGS revealed a de novo 12-kbp inversion that involved the first two exons of *BRPF1* and *CPNE9* in both probands confirmed by PCR (Fig. 4c). Disruptions of *BRPF1* are associated with a specific form of intellectual disability consistent with the proband’s phenotype (OMIM: 617333). Use of LRS data allowed the authors to determine that the inversion breakpoints mapped to a (TA)_n_ simple repeat element and to a mammalian-wide interspersed repeat (MIR) element, which is part of the short interspersed nuclear element (SINE) family. Due to the absence of indels at the junctions or sequence homology, nonhomologous end joining was proposed as the likely mechanism. The variant was deemed to be pathogenic because of the association of variants in *BRPF1* with intellectual disability. It is worth noting that the SV caller used in this study (PBSV) miscalled this 12-kbp inversion (called twice as a deletion and as an insertion of different size) and further examination of reads alignment was required to characterize the variant. Complete sequence assembly of the phased haplotype in conjunction with Strand-seq would have likely fully resolved the pathogenic variant of interest [10].

The use of LRS on the ONT platform has become more prevalent in the clinical research community because of lower startup costs, lower materials costs, faster turnaround times, and greater flexibility (Table 1). There is a wide range of devices that vary in throughput (Table 1) ideal a variety of clinical (and field) applications where either whole-genome or targeted sequencing may be performed without the expense or footprint of large machines. The ONT platform is particularly nimble with respect to turnaround time [27, 28]. For example, Watson and colleagues used a targeted approach to evaluate an individual with suspected Meckel-Gruber syndrome (MKS), an autosomal recessive ciliopathy [12]. The condition is typically perinatally lethal and presents with cranial abnormalities, polydactyly, and other congenital malformations. The DNA of a fetus suspected to be affected by MKS initially underwent an SRS assay targeting 223 genes associated with pediatric neurological disorders, including 29 genes associated with MKS and Joubert syndrome. Two likely pathogenic heterozygous variants (1 SNV and 1 deletion) were identified in *TMEM231* with SRS. The long-range PCR product was subsequently sequenced using ONT and confirmed only the SNV. Moreover, T-LRS revealed a cluster of four SNVs absent in the SRS data. The same cluster was previously reported in the literature as the result of a gene conversion between *TMEM231* and a downstream pseudogene [138]. Sanger sequencing confirmed the gene conversion in the fetus (Fig. 4d). SRS data had incorrectly mapped the SNV cluster to the downstream pseudogene instead of *TMEM231*. This incorrect assignment essentially eliminated the SNVs from consideration and resulted in reduced coverage within the converted region of *TMEM231*, mimicking a heterozygous deletion. LRS analysis also revealed that the two variants identified were in trans and inherited from the healthy carrier parents, providing a good example of the additional information that LRS can provide in the clinical setting and how it may be used to overcome SRS mapping limitations.

Recent work has also shown high concordance between SVs identified by clinical testing and LRS. For example, Miller and colleagues evaluated 30 individuals with known SVs, using adaptive sampling on the ONT platform [15]. The authors reported 100% concordance with known SVs identified by clinical testing and LRS. In all eight individuals with known complex structural rearrangements, T-LRS identified the known aberrations and identified additional as well. They also showed that systematic evaluation of missing variant cases, or those with a single pathogenic variant in a gene associated with a recessive condition or no pathogenic variants found in suspected X-linked or dominant disorders, using LRS is high yield.

## Summary and concluding remarks

In this review, we focused on the utility and advantages of LRS with respect to clinical research and human health. In short, both PacBio and ONT offer a more complete view of human variation and identify disease-causing variants missed by evaluation with both clinical and research SRS pipelines. These technologies led to the first complete human genome sequence [4], threefold improved SV discovery [6, 7, 9], more complete RNA sequencing [108, 109, 125], and modified base characterization of the human genome [30, 94]. PacBio offers greater sequencing accuracy, comparable to that of SRS, while ONT provides longer reads (up to >2 Mbp), rapid turnaround, and direct RNA sequencing.

Advances in SRS technology have been driven by massive increases in parallelization per machine to decrease cost and increase throughput per human genome. LRS technology, in contrast, has been focused on increasing read length and sequence accuracy. The recent launch of Revio by PacBio, which promises a highly accurate sequence of a human genome for $1000 in materials per human sample, represents an important shift in strategy and will allow more researchers to access high-quality LR WGS data. We also anticipate similar improvements with ONT, including duplex sequencing and advances in chemistry and pore structure to improve sequencing accuracy and increase output. Duplex sequencing allows for the sequencing of both strands of DNA, resulting in an increase in accuracy, but potentially sacrificing output and thus coverage [139].

At present, the two LRS technologies appear to be complementary and useful for different purposes. It is, however, tempting to speculate when LR WGS might emerge as a single test for clinical samples. Despite being more expensive than SR WGS, LR WGS advantages are clear: improved variant discovery (particularly for SVs), physical phasing of genomes, simultaneous discovery of methylation differences and genetic variants without additional experiments, and the ability to reanalyze a single dataset based on clinical suspicion. It is, in principle, the most comprehensive test currently available as it has the potential to fully sequence resolve both maternal and paternal chromosomes of a patient. If de novo assemblies of patient genomes and their parents become routine, it fundamentally changes how variants are discovered. Instead of read-based discovery, parent-to-offspring comparison of fully resolved chromosomes can be made to discover genetic and epigenetic changes of both small and large effect (Fig. 2). As the disadvantages, including, cost, throughput, and computational overhead are resolved, LRS will become a more attractive option to human genetics researchers and clinicians alike.

Recent efforts have tried to build automated and standardized pipelines for the screening and analysis of SVs [66, 140]. Mitsuhashi et al*.* focused on the analysis of large-scale chromosomal rearrangements, while Miyatake et al*.* on the analysis of repeat expansions. Both strategies call variations from read alignment to a reference, use a small control dataset (27 and 33 individuals) to remove common benign variations, and provide a list of prioritized candidate SVs for further manual investigation. Some type of variants (e.g., small deletions in Mitsuhashi et al*.*) are removed by the filtering step or by design (Miyatake et al*.* workflow is a targeted approach to only study repeat expansion loci associated to disease) to focus on the variant type of interest. These two workflows are very useful to simplify downstream analysis but also highlight the need for dedicated pipelines per SV type and larger control datasets. In fact, a current limitation of LRS is data interpretation, particularly for SVs of unknown significance and ultra-rare variants. At the moment, large databases of LRS samples from population controls comparable to SRS samples do not exist; SRS databases such as gnomAD-SV [8] cannot be used to assess frequency of a particular variant and the tolerance to mutation of many of the genes being accessed for the first time are unknown (e.g., there are no pLI [probability of being loss-of-function intolerant] [141] or LOEUF [loss-of-function observed/expected upper bound fraction] [142] scores for duplicate genes). Also, it is extremely important to have all populations represented in control datasets to perform a thorough screening of common variations.

Several efforts are underway to begin to characterize the normal pattern of variation (Table 3) in the general population using LRS. We expect that the increasing use of LRS in clinical studies and matched population controls will deepen our knowledge and interpretation of SVs and the decrease in sequencing cost will lead to progressively larger studies, as already seen for some circumscribed populations [143]. Together, these efforts will lead to improved outcomes for individuals with suspected Mendelian conditions who today remain unsolved after comprehensive evaluation, uncover novel biological processes that may be amenable to directed therapies, and allow development of improved clinical tests to reduce the time required to make precise genetic diagnoses.

**Table 3:** Consortia using long-read sequencing

**Project / consortium**	**Description**	**Estimated samples involved for LRS**	**Sequencing technology employed**
HGSVC	The Human Genome Structural Variation Consortium aims to create a high-quality map of human structural variation analyzing the genomes of individuals from different human populations and develop new discovery/analysis methods.	69+	PacBio, ONT
HPRC	The Human Pangenome Reference Consortium aims to develop a novel genome reference able to include all human genome variations and represent the full diversity of the human populations.	350+	PacBio, ONT
GREGoR	The GREGoR Consortium aims to substantially increase the number of Mendelian disorders with a known genetic cause focusing on the study of clinical cases.	500+	PacBio, ONT
ONT 1000 Genomes Project	The ONT 1000 Genomes Project aims to create a comprehensive genomic dataset of a large, diverse group of persons and provide an extensive catalog of structural variations.	500+	ONT
*All of Us*	The *All of Us* project aims to create a collection of genomic data from a large number of individuals in the United States.	12,000+	PacBio, ONT

## Supplementary Information

**Additional file 1:** **Table S1.** Additional examples of disease-associated variations resolved by LRS.

## Data Availability

Not applicable.

## Abbreviations

ACMG: American College of Medical Genetics
CATCH: Cas9-Assisted Targeting of Chromosome segments
CCS: Circular consensus sequencing
cDNA: Complementary DNA
CLR: Continuous long-read
dNTPs: Deoxynucleoside triphosphates
ES: Exome sequencing
GPU: Graphical processing units
HGSVC: Human Genome Structural Variation Consortium
Hi-C: High-throughput capture chromatin conformation
HiFi: High-fidelity
HMW: High molecular weight
HPRC: Human Pangenome Reference Consortium
Iso-Seq: Isoform sequencing
LITS: Large-insert targeted sequencing
LOEUF: Loss-of-function observed/expected upper bound fraction
LR WGS: Long-read whole-genome sequencing
LRS: Long-read sequencing
MKS: Meckel-Gruber syndrome
MLPA: Multiplex ligation-dependent probe amplification
MIR: Mammalian-wide interspersed repeat
ONT: Oxford Nanopore Technologies
PacBio: Pacific Biosciences
PAV: Phased assembly variant
PFGE: Pulsed field gel electrophoresis
pLI: Probability of being loss-of-function intolerant
PTLS: Potocki-Lupski syndrome
RIN: RNA integrity number
SINE: Short interspersed nuclear element
SMRT: Single-molecule, real-time
SNV: Single-nucleotide variant
SR WGS: Short-read whole-genome sequencing
SRS: Short-read sequencing
Strand-seq: Single-cell strand sequencing
SV: Structural variation
T2T: Telomere-to-telomere
T-LRS: Targeted long-read sequencing
T-SRS: Targeted short-read sequencing
ZMW: Zero-mode waveguide
5mC: 5-Methylcytosine
