# Supplementary material for: Applications of long-read sequencing to Mendelian genetics
Source: Genome Med. 2023 Jun 14;15:42. doi: 10.1186/s13073-023-01194-3 (PMC10266321; doi:10.1186/s13073-023-01194-3)
Supplement: Supplementary file 1 — Additional file 1: Table S1. Additional examples of disease-associated variations resolved by LRS. [file 13073_2023_1194_MOESM1_ESM.pdf]

Table S1. Additional examples of disease-associated variations resolved by LRS.

Variant Class	Associated Disease	Long-Read Sequencing Technology	Previous Approaches	Details	Citation
Single-nucleotide variant	Meckel-Gruber syndrome	T-LRS ONT	T-SRS	Identification of SNVs and characterization of a gene conversion event	(12)
Trinucleotide repeat expansion	Myotonic Dystrophy Type 1	T-LRS HiFi	-	Multiplex sequencing assay of amplicons containing a trinucleotide repeat expansion	(26)
Structural variations	Acute myeloid leukemia	ONT WGS	Karyotyping, RNA-sequencing	Breakpoint characterization of a previously identified translocation and characterization of an inferred translocation	(129)
	Usher syndrome Type 1	ONT WGS, T-LRS ONT	T-SRS	Identification and characterization of a large pathogenic inversion	(11)
	Different Mendelian conditions	T-LRS ONT	Karyotyping, microarrays, T-SRS, SR WGS	Identification and characterization of pathogenic SVs	(15)
	Neurodevelopmental disorders	HiFi WGS	SR WGS	Identification of large, complex, likely pathogenic SVs	(14)
	Hereditary cancer	T-LRS ONT	T-SRS	Characterization of mobile element insertions	(130)
	Intellectual disability	HiFi WGS	ES	Identification of a pathogenic inversion	(16)
	Breast cancer	T-LRS HiFi	-	Characterization of *BRCA1/2* loci and identification of a novel SINE-VNTR-Alu retrotransposon insertion	(63)
	Muscular dystrophy	ONT WGS	ES	Identification of pathogenic SVs and SNVs	(23)
	Microphthalmia, anophthalmia, and coloboma spectrum	ONT WGS	ES, microarrays	Characterization of a complex chromosomal rearrangement	(24)
